# Classification of outcomes in antimalarial therapeutic efficacy studies with Aster

**DOI:** 10.1128/aac.01411-25

**Published:** 2026-04-13

**Authors:** Inna Gerlovina, Sophie Berube, Jessica Briggs, Kathryn Murie, Maxwell Murphy, Amy Wesolowski, Bryan Greenhouse

**Affiliations:** 1 EPPIcenter Research Program, Division of HIV, ID and Global Medicine, Department of Medicine, University of California 8785, San Francisco, California, USA; 2 Department of Biostatistics, University of Florida 3463, Gainesville, Florida, USA; 3 Department of Epidemiology, Johns Hopkins Bloomberg School of Public Health 25802 , Baltimore, Maryland, USA; The Children's Hospital of Philadelphia, Philadelphia, Pennsylvania, USA

**Keywords:** malaria, *Plasmodium falciparum*, molecular correction, genotyping, statistics, recrudescence, antimalarial

## Abstract

Reliable assessment of antimalarial drug efficacy is crucial for an effective response to emerging drug resistance, and therapeutic efficacy studies (TESs) are the primary means of estimating *in vivo* efficacy. The accuracy of such estimates rests on correctly classifying recurrent infections developed during follow-up as recrudescences or new infections. Genotyping is used to guide classification, but polyclonal infections and alleles matching by chance make classification challenging, especially in high transmission settings. Match-counting algorithms currently recommended by the World Health Organization are unreliable and produce biased results, necessitating the development of principled statistical approaches. Modern genotyping methods, such as multiplexed amplicon sequencing, hold great potential for resolving recurrences and motivate the need for corresponding statistical methods able to utilize the rich data they provide. We propose an Adaptive Statistical framework for Therapeutic Efficacy and Recrudescence (Aster) that delivers accurate and consistent results by explicitly incorporating the complexity of infection, population allele frequencies, and imperfect detection of alleles in minority strains. Using an identity-by-descent approach, Aster accounts for alleles matching by chance and for a background infection relatedness structure that can otherwise lead to misclassification. The flexible framework can also use external information, such as parasite density and performance characteristics of a genotyping panel. Using simulations, we show that Aster dramatically outperforms match-counting algorithms in a wide variety of transmission settings and demonstrates consistently balanced performance that improves with more informative genotyping panels. Aster is implemented in a fast, fully scalable, and user-friendly R software package, *asterTES,* and provides accurate estimates of treatment failure for TES with any type of genotyping data, facilitating reliable evaluation of drug efficacy and effective management of malaria.

## INTRODUCTION

Widespread availability of highly effective artemisinin combination therapies has greatly improved treatment for *Plasmodium falciparum* infections, the most fatal cause of malaria, and has contributed to a global reduction in malaria burden over the last few decades (1). Unfortunately, progress in malaria control has recently stalled, with over 600,000 malaria deaths estimated in 2023 (2). One of the largest threats to malaria control right now is the emergence of artemisinin partial resistance in sub-Saharan Africa, where >95% of malaria cases and deaths occur (3, 4). Treatment with a failing drug often results in partial clearance of infecting parasites, which then recrudesce, resulting in increased morbidity, mortality, and ongoing transmission of resistant parasites. Antimalarial efficacy is primarily evaluated through therapeutic efficacy studies (TESs). A TES or similar clinical trial of antimalarial efficacy assesses *in vivo* efficacy by enrolling patients with symptomatic malaria infections, administering directly observed drug therapy, and actively following these individuals to assess parasite clearance. Since recrudescence often occurs several weeks after therapy, extended follow-up times are required to assess for recurrent parasitemia. Importantly, the World Health Organization (WHO) recommends changing therapy if genotype-corrected failure (recrudescence) rates exceed 10%, making TES results a vital source of information to determine treatment policy (5).

In malaria endemic areas, TES participants can be reinfected during the long follow-up period. Measuring efficacy thus requires determining whether detection of asexual parasites during follow-up is due to an entirely new infection, which is not an indication of drug failure, or a recrudescence (possibly in combination with a new infection), suggesting true drug failure (5, 6). In areas with high malaria transmission, including most of sub-Saharan Africa, where artemisinin partial resistance is now emerging, people can be infected hundreds of times per year, and over 50% of study participants can develop new infections during follow-up (7, 8). The results of TES in these areas—and by extension treatment policy—can, therefore, be dramatically affected by the accuracy of outcome classification (9–12). The genotypes of parasites present in the blood prior to treatment (day zero [D0]) are compared with those present when recurrent parasitemia is first detected by microscopy (day of recurrence [DR]); matching parasite genotypes between D0 and DR suggest recrudescence, while the DR genotypes that are different from the ones on D0 suggest that a new infection has occurred (6). Since *P. falciparum* populations in sub-Saharan Africa exhibit high levels of genetic diversity, it is unlikely that an individual will, by chance, be reinfected with a genetically identical parasite (6, 13, 14).

In practice, biological and technical factors complicate the comparison, compromising the accuracy of TES results (4, 15). *P. falciparum* infections frequently contain multiple genetically distinct strains, i.e., have a complexity of infection (COI) greater than 1 (9, 16). While genotyping can usually detect multiple alleles at each locus, it is not currently feasible to assign alleles across loci to underlying strains (i.e., the genotyping data are unphased). Since a recurrent infection would constitute a recrudescence event if it contains at least one persistent strain, regardless of the presence or absence of newly infecting strains, any matching alleles between D0 and DR at a given locus provide potential evidence of recrudescence. However, alleles from genetically distinct parasites can also match by chance, potentially resulting in misclassifying a new infection (i.e., an infection with no persistent pre-treatment strains) as a recrudescence; polyclonality exacerbates the issue by increasing the chance of matching (9, 10). Technical issues pose additional challenges for classification: misidentification of genotyping artifacts as additional alleles (i.e., false positive alleles) can produce non-existent matches, while alleles from strains at low frequencies in the blood might go undetected (i.e., false negative alleles), potentially masking matches that are due to recrudescence (12, 17, 18).

To address these challenges, the WHO recommends genotyping three diverse loci and assessing allele matches between D0 and DR samples (19). If these genotypes have at least one matching allele at all three loci, the recurrence is considered a recrudescence (“3/3 algorithm”) (20); otherwise, it is classified as a new infection. With this algorithm, however, undetected alleles in recrudescent strains could result in misclassifying such recurrences as new infections. To allow for imperfect sensitivity of genotyping methods, the “2/3 algorithm” has been proposed as an alternative, requiring only two of the three loci to have matching alleles. These match-counting algorithms are easy to implement, but they do not explicitly account for factors that can have a large effect on the presence of matching alleles not due to recrudescence, such as allele frequency, COI, and underlying relatedness structure in the local parasite population. Resulting misclassifications lead to estimates of drug failure that may be considerably biased by factors such as transmission intensity or the genotyping method used (4, 10, 11, 15).

In light of the limitations of match-counting algorithms, the need for statistical approaches to estimate therapeutic failure rate has become apparent. Such approaches can account for biological and technical factors in a principled way, reducing bias and providing measures of uncertainty. The first likelihood-based method, the CDC Bayesian algorithm (12), uses a Monte Carlo Markov chain approach and provides recurrence classification and posterior probability of recrudescence. More recently, another likelihood-based method, *PfRecur*, has been proposed (21); it provides a posterior probability of a recrudescence event and a posterior expected proportion of recrudescent clones. Both methods are designed for genotyping data based on length polymorphisms and account for incorrectly identified and undetected alleles, assigning the same probability of detection to every allele (12) or parasite clone (21). A different, nonparametric statistical approach (22) uses a null distribution of a genetic dissimilarity statistic between two non-recrudescent infections, approximated by an empirical distribution of pairwise dissimilarity between D0 infections, to classify recurrences. This method can be used with a dissimilarity measure of the user’s preference.

Continuing development of amplicon sequencing methods, which likely have advantages over length polymorphisms in accuracy, reproducibility, accessibility, throughput, and cost (23–28), offers potential for more reliable assessment of therapeutic failure rates. Higher numbers of genotyped loci and more reliable detection of alleles from minority strains in complex infections can improve the accuracy of estimation. The increasing generation of amplicon sequencing data motivates the need for corresponding statistically rigorous approaches that are flexible, fully scalable, able to utilize various types of information, and are easy to use. Here, we propose a novel statistical approach, the Adaptive Statistical framework for Therapeutic Efficacy and Recrudescence (Aster), that performs recurrence classification along with failure rate estimation, providing inference based on an empirical Bayes approach. Aster explicitly accounts for population allele frequencies, COI, imperfect allele detection, and an underlying relatedness structure in the local parasite population (here referred to as “background relatedness”). The model approaches recrudescence through the concept of identity by descent (IBD), which allows it to track recrudescent and minor clones across loci. This approach also provides a natural way to account for the possibility of being reinfected with clones that are genetically related to the ones in a D0 sample, which can otherwise lead to misclassification (29). Aster is implemented in the software package *asterTES* (30), which is fast, numerically stable, scalable, and user-friendly. It uses efficient combinatorial algorithms to process unphased data.

First, we present the overall framework, then evaluate Aster’s performance against match-counting algorithms across a variety of genotyping methods and epidemiological settings using simulations. We conclude by discussing practical applications of the method.

## MATERIALS AND METHODS

### Framework

Aster is designed to infer drug failure rates in antimalarial therapeutic efficacy studies. The adaptive framework consists of two main components: (i) the genetic data generating model and (ii) the detection model. We assume independence between the corresponding random processes. Each of the models can be adapted to different settings and can utilize external information, allowing for flexibility in practical applications. Overlaying these models is an observation mechanism, which reflects the fact that phased genotype data are not usually directly observed; this mechanism is represented by a deterministic function. At the top level, the framework addresses recrudescence, allowing both strain- and person-specific definitions. Here, we present the latter, the most common approach where recrudescence is defined as an event where at least one strain from the D0 infection is recrudescent. In this case, the therapeutic failure rate is the probability that an individual has a recrudescence event following therapy. First, we describe the framework components and observation mechanism for a single sample, not considering recrudescence, then expand them to a pair of samples from the same individual. The relationship between recrudescence and IBD is a part of the genetic model and is described in the section “Pair of samples and recrudescence.”

#### Single sample

##### Genetic data generating model

**Fig 1:**
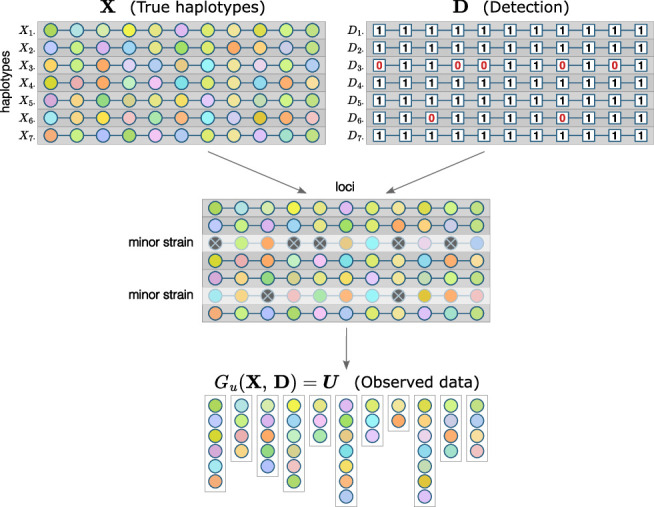
Framework components for a single sample. Each row of X and D matrices represents a strain, each column—a locus. Colors inside circles represent alleles: for each locus, the same color indicates the same allele; multiple strains can have the same allele at a given locus. Minor strains are not fully detected, which is indicated with crossed-out dark gray circles, but an allele that is undetected in one strain can still be detected at a locus if it is present in another strain. Observed data for each locus are a set of detected unique alleles.


Let X be a n×L matrix of random variables, where n is COI, L is the number of loci, rows $X_{i\cdot }=(X_{i1},\ldots ,X_{iL})$ represent distinct parasite genotypes, and columns $X_{\cdot l}=(X_{1l},\ldots ,X_{nl})$ represent alleles at each locus (Fig. 1). X can be thought of as phased genotype data for a single infection. Each $X_{il}$ has a categorical distribution with values in a set $A_{l}=\{a_{l1},\;\ldots ,\;a_{lK_{l}}\}$ of possible alleles at locus l and corresponding probabilities—population allele frequencies $\pi (a_{lk})$. $X_{il}$ are assumed to be independent, which implies no linkage disequilibrium, perfect mixing of parasite strains, and no intrahost relatedness.

$$P(X)=\prod_{i=1}^{n}\prod_{l=1}^{L}\pi (X_{il}).$$

##### Detection model

In a polyclonal infection, parasite strains that are present in a sample in smaller proportions (minor strains) might be harder to detect during genotyping, and for these strains, alleles at some loci might not be detected. Let D be a binary n×L matrix that represents detection: if $D_{il}=1$, an allele in strain $X_{i\cdot }$ at locus l has been detected, and if $D_{il}=0$, it has not been detected (Fig. 1). $D_{il}$ are independent random variables, $D_{il}∼Bernoulli(p_{il})$, where $p_{il}$ is the probability of detection.


$$P(D)=\prod_{i=1}^{n}\prod_{l=1}^{L}p_{il}^{D_{il}}\;(1-p_{il}{)}^{1-D_{il}}.$$

In addition to strain proportion, $p_{il}$ can depend on panel characteristics, overall parasite density, COI, and other factors. In many cases, it is reasonable to assume the same probability for alleles from a strain to be detected at any locus ($p_{i1}=\ldots =p_{iL}$), but it is possible that some markers can be more sensitive, in which case these probabilities will vary from locus to locus. Major strains can be assumed to be fully detected ($p_{jl}=1\;\forall \;l$ for some major strain j). There might also be completely undetected strains that are not present in the sample; this special case of $p_{kl}=0\;\forall \;l$ for an undetected strain k can be important for classification if that strain is also recrudescent. While the Aster framework formally allows such a scenario, incorporating it would require careful consideration (and possibly extending the framework) to avoid classification bias and/or extremely high uncertainty (see Section S.4 in the supplemental material).

##### Observation mechanism

When genetic data for polyclonal infections are unphased, observed data consist of unordered lists of unique alleles detected at each locus. Let $U=(U_{1},\;\ldots ,\;U_{L})$ be a sequence of sets that represents the observed data. U is fully determined by matrices X and D, and the observation mechanism can be represented by a deterministic function $G_{u}$ that maps haplotype and detection matrices to a sequence of sets of detected unique alleles (Fig. 1). To define $G_{u}$, we first consider a single locus. Let $g_{u}((a_{i}{)}_{i=1}^{n},\;(b_{i}{)}_{i=1}^{n}))$ be a function that maps two sequences of the same length, the second of which is binary, to a set $U=\{a_{i}:\;b_{i}=1\}$ [$(b_{i}{)}_{i=1}^{n}$ represents detection and indicates which elements of $(a_{i}{)}_{i=1}^{n}$ are included in U]. Applying $g_{u}$ to paired columns of X and D, we get $U=(g_{u}(X_{\cdot 1},\;D_{\cdot 1}),\;\ldots ,\;g_{u}(X_{\cdot L},\;D_{\cdot L}))$*,* full observed data for a single sample. Thus, $G_{u}$ is defined as $G_{u}(X,\;D)\equiv (g_{u}(X_{\cdot l},\;D_{\cdot l}){)}_{l=1}^{L}$. Note that if an allele from a minor strain i at locus l has not been detected ($D_{il}=0$), it can still belong to $U_{l}$ if it is present and has been detected in other strains.

##### Probability of observed genotyping data

Since $X_{il}⊥D_{jm}\;\forall \;i,j,l,m$*,* the probability of observed data U for an infection with n distinct clones can be written simply as


(1) $$P(U)=\sum_{X,D:G_{u}(X,D)=U}\;P(X)P(D).$$

The summation in equation 1 is taken over all the combinations of full genetic and detection matrices that are compatible with observed data, thus making this direct approach computationally unfeasible for most cases and generally unscalable; including a second sample combined with recrudescence adds another layer of complexity. While Aster uses other approaches described below to efficiently calculate the likelihood, it is a useful reference to what exactly needs to be calculated. Equation 1 does not explicitly exclude matrices with repeated rows, and there is a non-zero probability that two unrelated strains are identical by state at all the loci in a sequencing panel. However, if the panel is informative (i.e., has a sufficient number of diverse loci), this probability is small; in addition, if $|U_{l}|=n$ for at least one locus l, which is often the case in practice, as most COI estimation methods for multiallelic data would yield estimates that are no greater than $\max(|U_{1}|,\;\ldots ,\;|U_{L}|)$*,* no matrices with repeated rows will be compatible with U.

Using a single sample, we introduce some of the concepts of Aster implementation. First, since loci can be considered separately (while keeping in mind that for both recrudescence and detection, strains/rows need to be tracked across the loci by keeping their indices fixed), we can switch the order of the sum and the product, greatly reducing the number of combinations:


$$P(U)=\;\;\;\;\;\;\sum_{\begin{array}{c} X,\;D:\\ G_{u}(X,\;D)=U \end{array}}\;\;\;\;\;\;P(X)P(D)=\;\;\;\;\;\;\sum_{\begin{array}{c} X,\;D:\\ G_{u}(X,\;D)=U \end{array}}\;\;\left[\prod_{l=1}^{L}P(X_{\cdot l})\right]\left[\prod_{l=1}^{L}P(D_{\cdot l})\right]\;\;=\;\prod_{l=1}^{L}\;\;\;\;\;\sum_{\begin{array}{c} X_{\cdot l},\;D_{\cdot l}:\\ g_{u}(X_{\cdot l},\;D_{\cdot l})=U_{l} \end{array}}\;\;\;\;\;\;\;\;P(X_{\cdot l})\;P(D_{\cdot l}).$$

Next, we define a function that allows us to calculate the probability of a set of unique alleles in a number of strains at a given locus without explicitly allocating these alleles to specific strains. Let $P_{u}(U,\;n)$ be a function that calculates the probability that a sequence of length n has all the elements of set U with corresponding probabilities and only elements from set U: for a sequence $Z_{1},\;\ldots ,\;Z_{n}$ of independent identically distributed categorical random variables,


(2) $$P_{u}(U,\;n)=P\left(Z_{i}\in U\;\forall \;i\;\text{ and }\;\forall \;a\in U\;\exists \;j:\;Z_{j}=a\right).$$

Set $P_{u}(\emptyset ,\;0)=1$ (no elements in a zero-length sequence); also note that $P_{u}(U,\;n)=0$ if |U|>n and $P_{u}(\emptyset ,\;n)=0$ if n>0. In Aster, elements of U are alleles, their corresponding probabilities are population allele frequencies, and n is the number of strains to which alleles in U belong. Section S.1 in the supplemental material provides two efficient ways to calculate $P_{u}(U,\;n)$.

To incorporate missingness, recall that $P(D_{il}=1)=p_{il}$. If $D_{il}=0$, i.e., an allele in strain i at locus l is not detected, $X_{il}$ is unknown and can be any allele $a\in A_{l}$; consequently, if alleles from all the other strains j≠i are detected, $P(U_{l}\;|\;n,D_{il}=0)=P_{u}(U_{l},\;n-1)$ (see Section S.2 in the supplemental material for details). Then, for a sample with COI of n and a single minor strain i,


(3) $$\begin{array}{lrlr} & P(U_{l}\;|\;n) & =P(U_{l}\;|\;n,D_{il}=1)\;P(D_{il}=1)+P(U_{l}\;|\;n,D_{il}=0)\;P(D_{il}=0) & \\ & & =p_{il}\;P_{u}(U_{l},\;n)+(1-p_{il})\;P_{u}(U_{l},\;n-1) & \end{array}$$

for locus l.

In general, for any set of minor strains,


(4) $$P\left(U_{l}|n\right)=\sum_{D_{\cdot l}\in B^{\left(n\right)}}\left[\prod_{i:D_{il=1}}p_{il}\right]\left[\prod_{j:D_{jl}=0}\left(1-p_{jl}\right)\right]P_{u}\left(U_{l},\sum_{i=1}^{n}D_{il}\right),$$

where $B^{\left(n\right)}$ is a set of all the possible binary sequences of length n (note that the terms with $D_{\cdot l}$ such that $\sum_{i=1}^{n}D_{il}<|U_{l}|$ are equal to 0).

### Pair of samples and recrudescence

Recrudescence can be viewed at a person level (i.e., an individual in a study) or, alternatively, considering that many infections are polyclonal, at a strain level. Commonly, when therapeutic failure rates are estimated, a failure is defined on a person level—even if multiple strains in a single infection have recrudesced. Adopting this approach, we consider a case where at least one of the D0 strains has not completely cleared and is present in the DR sample (regardless of the presence or absence of newly acquired strains) to be a recrudescence event. Let $R_{j}$ be a binary random variable representing recrudescence for individual j, where $R_{j}=1$ indicates a recrudescence event, and let θ be a population-level failure rate. In TES, individual responses to therapy are commonly considered to be independent, so we assume $R_{j}∼Bernoulli(\theta )$, j=1,…,N, where N is the number of individuals in the study, to be independent and identically distributed.

#### Genetic data generating model

*Let* X and Y be $n_{x}\times L$ and $n_{y}\times L$ matrices representing D0 and DR infections from the same individual. We assume that newly infecting clones are acquired independently from recrudescence and, consequently, that recurrence of parasitemia can be a result of a new infection, a recrudescence, or both. If Y has recrudescent strains, the rows representing them are identical to the corresponding rows in X, while entries $Y_{jl}$ in the rows of Y that represent newly infecting strains (if any) are drawn from the same distribution as $X_{il}$. Since the order of the strains within an infection does not matter, we order them in such a way that recrudescent strains occupy the top rows of both matrices and their indices match in X and Y, e.g., if an individual has two recrudescent strains, then $X_{1l}=Y_{1l}\;\;\forall \;l$ and $X_{2l}=Y_{2l}\;\;\forall \;l$.

We approach modeling alleles in recrudescent strains by considering identity by descent. A recrudescent strain i is the same in both samples, and it can be stated that $X_{il}$ and $Y_{il}$ are IBD at all loci. Let $IBD_{il}$ be an indicator that $X_{il}$ and $Y_{il}$ are IBD. Then, using population allele frequencies $\pi (a_{lk})$,


$$\;\;\;\;\;\;\begin{array}{r} P(X_{il},\;Y_{il}\;|\;IBD_{il}=1)=\left\{ \begin{array}{ll} \pi (X_{il}) & \text{ if }X_{il}=Y_{il}\\ 0 & \text{ otherwise } \end{array}\right., \end{array}$$

$$\;\;\;\;\;\;P(X_{il},\;Y_{il}\;|\;IBD_{il}=0)=\pi (X_{il})\;\pi (Y_{il}).$$

For a pair of strains $X_{i\cdot },\;Y_{i\cdot }$, let $R_{i}^{\prime }$ be an indicator that strain i is recrudescent (we want to distinguish a strain-specific variable $R_{i}^{\prime }$ from R, a person-level recrudescence). If $R_{i}^{\prime }=1$, $IBD_{il}=1\;\forall \;l$; for the reverse to be true, we would need to assume that a person cannot be reinfected with strains that are related to the ones present at D0. That is not necessarily the case, and a population can have a non-zero level of background relatedness $r_{bg}$, which we define as a probability that a newly infecting strain and an originally present strain are IBD at a given locus. Then


$$\begin{array}{rl} & P\left(IBD_{il}=1|R_{i}^{\prime }=1\right)=1\\ & P\left(IBD_{il}=1|R_{i}^{\prime }=0\right)=r_{bg}. \end{array}$$

#### Detection model

Let $D_{x}$ and $D_{y}$ be $n_{x}\times L$ and $n_{y}\times L$ binary matrices representing detection for X and Y, with corresponding probabilities of detection $P(D_{x,il}=1)=p_{x,il}$ and $P(D_{y,il}=1)=p_{y,il}$. In addition to independence between full haplotype data and detection, we assume independence between detection in two samples $D_{x,il}⊥D_{y,jm}\;\;\forall \;i,j,l,m$ as well as independence between detection and recrudescence/IBD status: $D_{x,il}⟂IBD_{il}$ and $D_{y,il}⊥IBD_{il}$.

#### Observation mechanism

We denote observed data for samples from D0 and DR by $U_{x}=(U_{x,1},\;\ldots ,\;U_{x,L})=G_{u}(X,\;D_{x})$ and $U_{y}=(U_{y,1},\;\ldots ,\;U_{y,L})=G_{u}(Y,\;D_{y})$, respectively.

#### Likelihood

In general, for a failure rate θ,


(5) $$L\left(\theta ;U_{x},U_{y}\right)=\theta P\left(U_{x},U_{y}|R=1\right)+(1-\theta )P\left(U_{x},U_{y}|R=0\right)$$

$P(U_{x},U_{y}\;|\;R=0)=P(U_{x})\;P(U_{y})$*,* but to derive $P(U_{x},U_{y}\;|\;R=1)$*,* we need to consider strain-level recrudescence and look at probabilities with given sets of indices for minor and recrudescent strains (recall that recrudescent strain indices should match in X and Y). We start with a base case example where a possibly recrudescent strain is also a minor strain in both samples, then proceed to the general case; for derivations and special cases, see Section S.2 in the supplemental material. Suppose there is a single strain i=1 that might be recrudescent, a single minor strain $X_{1\cdot }$ in the D0 sample and a single minor strain $Y_{1\cdot }$ in the DR sample; also assume that strains $Y_{k\cdot }$, k>1, if any, are not related to any strains in the D0 sample. Then,


$$\begin{array}{rlrlrlrl} P\left(U_{x},U_{y}\right) & = & & \theta & & \prod_{l=1}^{L} & & \;\;P\left(U_{x,l},U_{y,l}|IBD_{1l}=1\right)\\ & +(1- & & \theta ) & & \prod_{l=1}^{L} & & [P\left(U_{x,l},U_{y,l}|IBD_{1l}=1\right)r_{bg}\\ & & & & & & & +P\left(U_{x,l},U_{y,l}|IBD_{1l}=0\right)\left(1-r_{bg}\right)] \end{array}$$

(see Section S.3 in the supplemental material).

To find $P(U_{x,l},U_{y,l}\;|\;IBD_{1l})$*,* we need to account for all four combinations of $D_{x,1l}$ and $D_{y,1l}$ with corresponding conditional probabilities $P(U_{x,l},U_{y,l}\;|\;D_{x,1l},D_{y,1l},IBD_{1l})$:


$$\begin{array}{rlrl} P\left(U_{x,l},U_{y,l}|n_{x},n_{y},IBD_{1l}=1\right)= & \;p_{x,1l}\;p_{y,1l} & & P_{xy}^{\left(1\right)}\left(U_{x,l},U_{y,l},n_{x},n_{y}\right)\\ & +\;p_{x,1l}\left(1-p_{y,1l}\right) & & P_{u}\left(U_{x,l},n_{x}\right)\;\;\;P_{u}\left(U_{y,l},n_{y}-1\right)\\ & +\left(1-p_{x,1l}\right)\;p_{y,1l} & & P_{u}\left(U_{x,l},n_{x}-1\right)P_{u}\left(U_{y,l},n_{y}\right)\\ & +\left(1-p_{x,1l}\right)\left(1-p_{y,1l}\right)\; & & P_{u}\left(U_{x,l},n_{x}-1\right)P_{u}\left(U_{y,l},n_{y}-1\right), \end{array}$$

where


(6) $$\begin{array}{rl} P_{xy}^{\left(1\right)}\left(U_{x},U_{y},n_{x},n_{y}\right)=\sum_{a\in U_{xy}}\pi (a) & \left[P_{u}\left(U_{x},n_{x}-1\right)+P_{u}\left(U_{x}\setminus \{a\},n_{x}-1\right)\right]\\ & \times \left[P_{u}\left(U_{y},n_{y}-1\right)+P_{u}\left(U_{y}\setminus \{a\},n_{y}-1\right)\right], \end{array}$$

$U_{xy}=U_{x}\cap U_{y}$, and a set difference U∖{a} is a set U without an element a. $P_{xy}^{\left(1\right)}(U_{x},U_{y},\;n_{x},n_{y})$ is a function that accounts for a single recrudescent strain between two samples using equation 2. When $IBD_{1l}=0$,


$$\begin{array}{c} P(U_{x,l},U_{y,l}\;|\;n_{x},n_{y},IBD_{1l}=0)=P(U_{x,l}\;|\;n_{x})\;P(U_{y,l}\;|\;n_{y}), \end{array}$$

where $P(U_{x,l}\;|\;n_{x})$ and $P(U_{y,l}\;|\;n_{y})$ are calculated as in equation 3.

In general, to accommodate any number and combination of possibly recrudescent strains, IBD pairs of strains, and minor strains, whether recrudescent or not, in both samples, we combine equation 4 with the generalized conditional probability, which is the key component of the likelihood:


$$P\left(U_{x,l},U_{y,l}|D_{x,\cdot l},D_{y,\cdot l},IBD_{\cdot l}\right)=P_{xy}^{\left(m\right)}\left(U_{x,l},U_{y,l},\sum_{i=1}^{n_{x}}D_{x,il},\sum_{j=1}^{n_{y}}D_{y,jl}\right),$$

where $IBD_{\cdot l}$ is a sequence $(IBD_{il}{)}_{i=1}^{min(n_{x},n_{y})}$*,* $m=\sum_{i=1}^{min(n_{x},n_{y})}D_{x,il}\;D_{y,il}\;IBD_{il}$, and


(7) $$P_{xy}^{\left(m\right)}\left(U_{x,l},U_{y,l},n_{x},n_{y}\right)\;=\;\;\;\;\sum_{U^{\prime }\;\subseteq U_{xy}}\;\;\;\;P_{u}\left(U^{\prime }\;,m\right)\;\;\;\sum_{U^{\prime \prime }\subseteq U^{\prime }}\;\;\;\;P_{u}\left(U_{x,l}\setminus U^{\prime \prime }\;,n_{x}\;-\;m\right)\;\;\;\sum_{U^{\prime \prime \prime }\subseteq U^{\prime }}\;\;\;\;P_{u}\left(U_{y,l}\setminus U^{\prime \prime \prime }\;,n_{y}\;-\;m\right)$$

Terms with $|U^{\prime }|>m$ in equation 7 are equal to 0 since P(U,n)=0 for |U|>n. Note that


(8) $$P_{xy}^{\left(0\right)}(U_{x,l},U_{y,l},n_{x},n_{y})=P_{u}(U_{x,l},n_{x})\;P_{u}(U_{y,l},n_{y}).$$

Alternatively, multisets can be used to calculate $P(U_{x,l},U_{y,l}\;|\;D_{x,\cdot l},D_{y,\cdot l},IBD_{\cdot l})$; this approach can be more efficient in some situations. A mixed radix system algorithm (31, 32) is used for the implementation of both approaches.

Equation 8 provides guidance for the treatment of missing data (no alleles detected at some loci in one or both samples). The proof that excluding such loci from the analysis does not introduce any bias can be found in Section S.4 in the supplemental material.

### Adaptivity

The framework can incorporate different types of available information and can be combined with other methods. Depending on the amount of external information and prior knowledge, additional assumptions and constraints can be used. Finally, it can accommodate different parameters of interest and produce a corresponding output, e.g., strain-level recrudescence.

Aster explicitly accounts for population allele frequencies and COI. Allele frequencies can be estimated from D0 samples, from a combination of D0 and new infections in recurrent samples, or can be provided from larger sample estimates with information borrowed from other studies; COI can be estimated using genetic data from a single sample or a collection of samples (33). Similarly, background relatedness can be estimated from D0 samples, or its distribution can be estimated from other sources; other information (e.g., household location, travel history, or use of interventions such as bed nets) can be available to estimate the probability of being reinfected with a related strain for a particular individual. The detection model has a flexible number of parameters, where the probability of detection can vary between samples, loci, and strains. They can depend on sample parasite density, COI, and characteristics of the genotyping panels such as sensitivity and diversity of the markers. Prior information on therapeutic failure rates can be accommodated (and might differ for different drug combinations) with a Bayesian approach; different treatments could be compared using different priors.

Instead of a fixed value or point estimate, a distribution of $r_{bg}$ might be available and can be incorporated into the analysis. Let $f_{bg}(r)$ be a probability density function (pdf) of $r_{bg}$. Then, for the likelihood of a failure rate θ,


(9) $$L\left(\theta ;U_{x},U_{y}\right)=\int_{0}^{1}P\left(U_{x},U_{y};\;\theta ,\;r_{bg}\right)\;f_{bg}\left(r_{bg}\right)\;dr_{bg}.$$

Similarly, distributions of detection probabilities can be incorporated. As an example, let the D0 sample have a single minor strain i with $p_{x,i1}=\cdots =p_{x,iL}=p_{x}$ and the DR sample have a minor strain j with $p_{y,j1}=\cdots =p_{y,jL}=p_{y}$; let $f_{x}(p)$ and $f_{y}(p)$ be pdf’s of $p_{x}$ and $p_{y}$, respectively. Then,


$$L\left(\theta ;U_{x},U_{y}\right)=\int_{0}^{1}\int_{0}^{1}P\left(U_{x},U_{y};\;\theta ,\;p_{x},p_{y}\right)\;f_{x}\left(p_{x}\right)\;f_{y}\left(p_{y}\right)\;dp_{x}\;dp_{y}.$$

Since true recrudescence status for recurrent samples is inferred and not known for certain, there are no simple conjugate priors for the Bayesian approach. However, the posterior distribution of θ can be calculated numerically:


(10) $$f_{\theta |U_{x},U_{y}}(s)=\frac{L\left(s;U_{x},U_{y}\right)f_{\theta }(s)}{\int_{0}^{1}L\left(u;U_{x},U_{y}\right)f_{\theta }(u)\;du},$$

where $f_{\theta }(s)$ and $f_{\theta |U_{x},U_{y}}(s)$ are prior and posterior pdfs of θ.

### Classification and inference

To classify a recurrence as a recrudescence or a new infection, we use the likelihood in equation 5. The likelihood is linear, and classification can be based on the sign of the slope of the likelihood, or, in other words, on comparing $P(U_{x},U_{y}\;|\;R=1)$ and $P(U_{x},U_{y}\;|\;R=0)$. Formally, for a person-level recrudescence,


$$P(U_{x},U_{y}\;|\;R=1)=P(U_{x},U_{y}\;|\;M=1)P(M=1)+\cdots +P(U_{x},U_{y}\;|\;M=n_{xy})P(M=n_{xy}),$$

where $n_{xy}=\min(n_{x},n_{y})$ and $M=\sum_{i=1}^{n_{xy}}R_{i}^{\prime }$ is the number of recrudescent strains. In most cases, however, it is sufficient to only consider a case with M=1: if multiple strains are recrudescent while M=1 is assumed, this only strengthens the evidence that R=1, and if there is more evidence for M=0 than for M=1, it is unlikely that multiple recrudescent strains are present. While it is theoretically possible for the slopes of the likelihood functions with and without M=1 assumption to have different signs [e.g., if P(M=1)≪P(M>1)], such a case is unlikely to represent a realistic scenario.

For classification inference, a useful measure would quantify the uncertainty of the individual classification, for which a posterior probability $P(R=1\;|\;U_{x},U_{y})$ is a natural choice (12). Let $A_{1}\equiv P(U_{x},U_{y}\;|\;R=1)$ and $A_{0}\equiv P(U_{x},U_{y}\;|\;R=0)$. Then,


$$P(R=1\;|\;U_{x},U_{y})=\frac{A_{1}P(R=1)}{A_{1}P(R=1)+A_{0}P(R=0)}.$$

Using an empirical Bayes approach, we borrow information from all the individuals in the study by first estimating 1−θ with the Kaplan-Meier survival estimator $\hat{S}(t_{end})$ (34), where $t_{end}$ is the end of the follow-up period, and then using that estimate as a prior probability:


$$P(R=1\;|\;U_{x},U_{y})=\frac{A_{1}(1-\hat{S}(t_{end}))}{A_{1}(1-\hat{S}(t_{end}))+A_{0}\;\hat{S}(t_{end})}.$$

In effect, this approach provides a formal way to compare $A_{0}$ and $A_{1}$, weighted by prior probabilities. If we assume a prior probability P(R=1)=0.5, $P(R=1\;|\;U_{x},U_{y})=A_{1}/(A_{1}+A_{0})$. The procedure used in this approach can be iterated: recurrence classification can be updated based on the posterior probability (e.g., adopting a 0.5 probability cut-off for the classification), leading to an updated failure rate estimate. For most practical applications, a single update will be sufficient for convergence.

In addition, available external information that would contribute to prior probability or distribution can be similarly incorporated (e.g., as shown in equation 10).

### Simulations

We used simulations to compare the performances of Aster and match-counting algorithms for various genotyping panels, transmission intensities, genotyping error scenarios, and background relatedness levels. Panel comparisons were based on the set of three length polymorphisms (LPs) currently recommended by the WHO (MSP-1, MSP-2, and a single microsatellite) (20) and a contemporary amplicon sequencing panel containing diverse microhaplotypes (MAD^4^HatTeR) (24). For the amplicon sequencing panel, subsets of loci for simulations were chosen based on their heterozygosity ranking using population allele frequencies obtained from the previously analyzed data sets from high transmission areas of sub-Saharan Africa (the heterozygosity ranking approach was used for simplicity and clarity of comparison; in practice, other factors, including allele detection sensitivity and specificity, might be taken into account as well). To reflect higher genotyping error rates of LP panels, we increased missingness (1 − probability of detection) twice and false positive rates five times compared to the amplicon sequencing panels (17, 35). Transmission intensity levels were reflected in sample COI and the number of new infections, where applicable. COIs were drawn from zero-truncated Poisson (ZTP) distributions, with D0 means of 1.2, 3, and 5 for low, moderate, and high transmission settings, respectively; since COIs for recurrent infections are usually lower, DR means were, respectively, decreased by a factor of 1.8 (bounded below by 1). Genotyping errors were included with a simple “per strain” detection model for missing (false negative) alleles and a “split” model for false positive alleles, where each correctly detected allele can give rise to additional falsely detected ones (31).

We used three types of simulation schemes with increasing complexity: simulated pairs of samples to evaluate classification performance, simulated studies with fixed proportions of recrudescences and newly acquired infections, and studies with simulated responses to treatment combinations using an established pharmacokinetic/pharmacodynamic (PK/PD) model (10, 36–38), modified to allow for cotransmission (multiple genotypes acquired from a single simulated mosquito inoculation). With the first simulation scheme, groups of three samples were generated: a D0 sample and two DR samples, with and without recrudescence. D0-DR pairs with recrudescence were used to obtain sensitivity, while D0-DR pairs without recrudescence were used for specificity. We simulated 10,000 such triads for each combination of transmission intensity, allele detection probability, background relatedness, and genotyping panel. With the second scheme, 1,000 studies were simulated for each combination. While the proportions of individuals with recrudescent strains and with newly infecting ones were fixed to remove variability due to the randomness of recrudescence events, the individuals to whom these strains were assigned were random, creating a random number of overlaps; thus, the numbers of total recurrences varied. The number of recrudescent strains for each individual was drawn from a ZTP distribution with a mean of 1/3 of their D0 COI. For PK/PD simulations, parasite density profiles were generated using model parameters suggested in reference 38 with adjusted half-maximal inhibitory concentrations and maximum kill rates to achieve therapeutic failure rates at or near 0.1 (39, 40). We used homogeneous Poisson processes to introduce new infection times with incidence per year of 6 for moderate and 12 for high transmission; the numbers of coinfecting strains were drawn from ZTP distributions with means dependent on transmission intensity (2.5 for moderate and 4 for high transmission). To mimic a TES protocol, we measured parasite density at routine time points (days 1–7, 14, 21, and 28); once a detection limit of $10^{8}$ parasites/body had been reached, a recurrence was confirmed.

Aster analysis was conducted using the *asterTES* R package (30). Where applicable, we used naive COI estimation with a locus rank and a probabilistic COI-adjusted method (31) to estimate population allele frequencies for Aster. Allele detection probabilities in PK/PD simulations were estimated from sample parasite density using empirical data from mixed-strain controls for model fitting. While false positive alleles are not formally included in the Aster framework, its implementation allows for processing genetic data with false positives. When the number of alleles detected at a locus is greater than or equal to the COI, we allow for a possibility that some alleles might be false positives and therefore some might still be undetected by including a tuning parameter regulating the probability of this scenario. A small value for such probability would not affect classification results unless there is overwhelming evidence of recrudescence at other loci, in which case this evidence would be weighed against that value, and recrudescence would not be ruled out. For classification using match-counting algorithms, we calculated a proportion of loci with matches to apply these algorithms to simulated data from panels with more than three loci. Matches in all the loci were required for recrudescence with the 3/3 algorithm and matches in at least two-thirds of the loci with the 2/3 algorithm; loci with no detected alleles in at least one of the samples were excluded from these calculations. Additional intermediate classification thresholds of 0.9 and 0.8 of match proportions were explored as well.

## RESULTS

### Classification performance

Aster uses a probabilistic approach for recurrence classification that explicitly accounts for biological and technical factors, including COI, allele frequency, background relatedness between infections, and imperfect detection of alleles. To evaluate its performance in identifying whether a DR infection is a recrudescence or a new infection, we first simulated pairs of infections with and without recrudescences. Using different genotyping panels and a range of transmission settings, allele detection probabilities, and background relatedness levels, we compared the sensitivity and specificity of Aster to those of the 3/3 and 2/3 match-counting algorithms.

**Fig 2:**
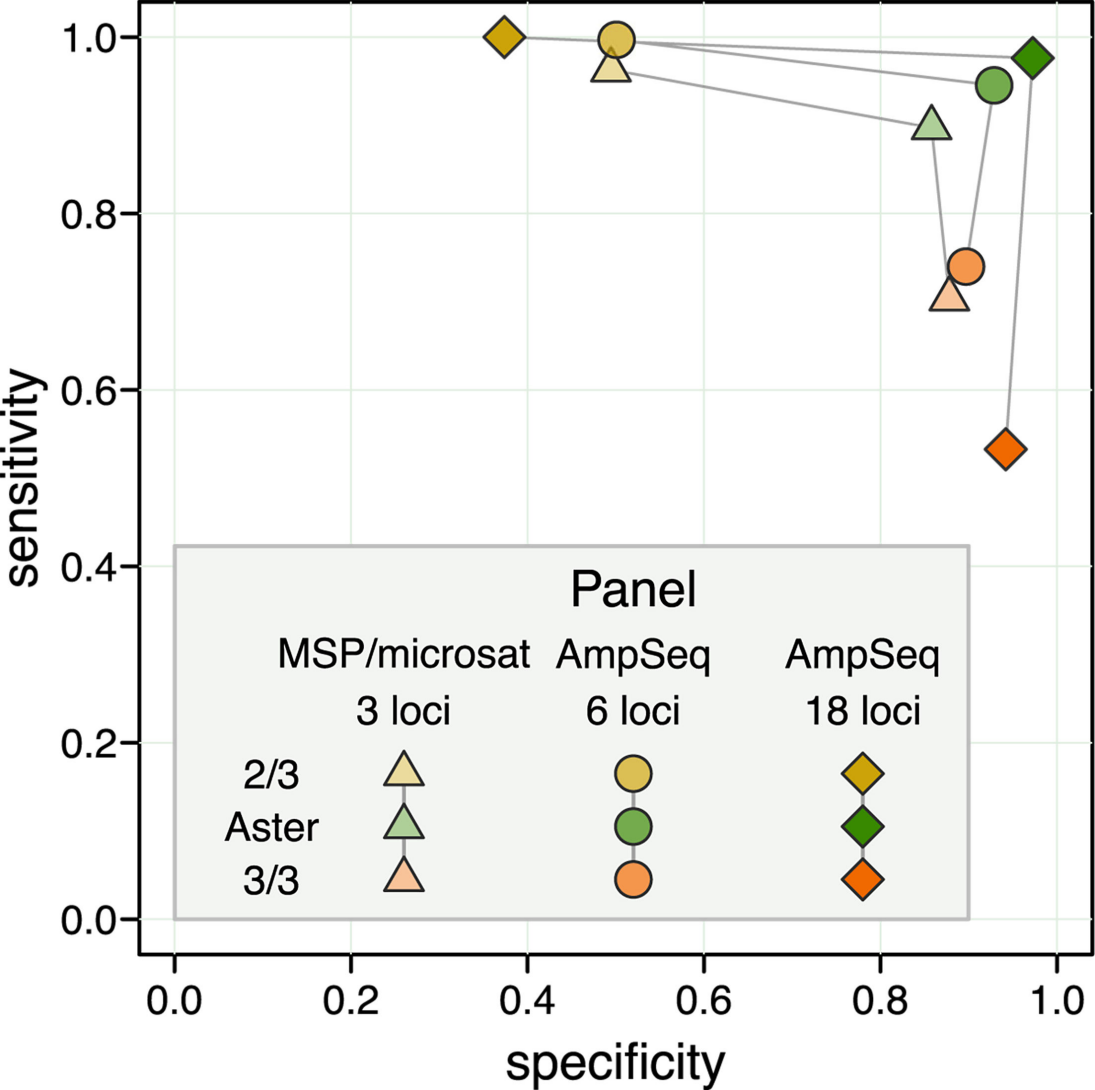
Sensitivity vs specificity for Aster, 3/3 algorithm, and 2/3 algorithm with three simulated genotyping panels: 3-locus length polymorphism (merozoite surface protein [MSP] 1, MSP-2, and a microsatellite) and 6 and 18-locus amplicon sequencing panels. A high transmission setting was used for these simulations, with a single recrudescent strain for each pair of samples and a partially detected recrudescent strain in a D0 sample; the probability of detection for that strain was set to 0.9 for all the loci in each panel. When applying match-counting algorithms to panels with >3 loci, the proportion of loci with allele matches among all loci with data in both samples was calculated; the value of 1 was required to classify a recurrence as a recrudescence by the 3/3 algorithm and of 2/3 or greater by the 2/3 algorithm.

In a simulated high transmission setting, Aster demonstrated balanced performance, exceeding 0.85 specificity and 0.9 sensitivity for all simulated genotyping panels and reaching near perfect classification with an 18-microhaplotype amplicon sequencing panel (Fig. 2). In contrast, the 3/3 match-counting algorithm had high specificity but much lower sensitivity, classifying many recurrences as new infections; the opposite was true for the 2/3 algorithm. The performance of match-counting algorithms worsened with increasing numbers of loci, reflecting the limiting nature of a mechanistic identity-by-state approach that does not benefit from additional information.

**Fig 3:**
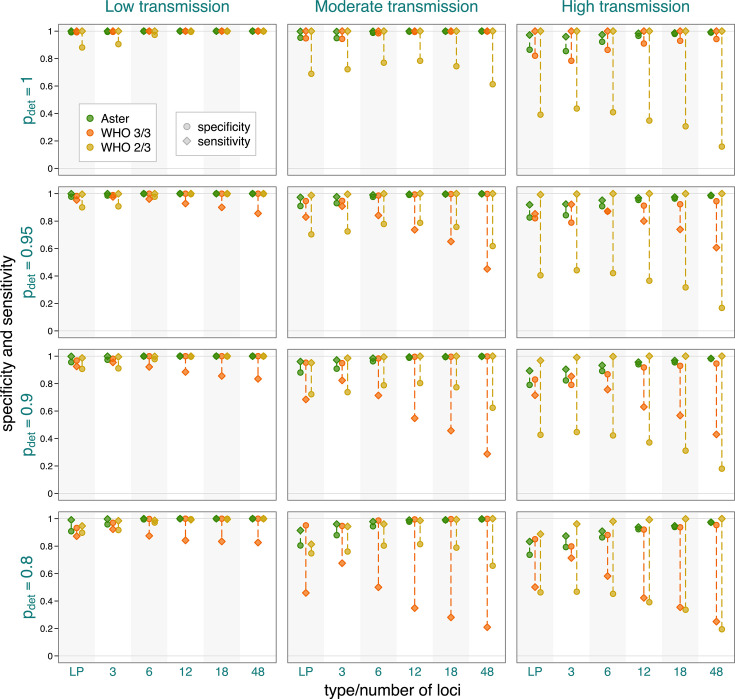
Sensitivity and specificity of Aster and match-counting algorithms across transmission intensity levels, genotyping panels, and detection probabilities ($p_{det}$). The panels presented are length polymorphisms (three loci) and MAD^4^HatTeR (varying number of loci). Dashed lines mark the difference between sensitivity and specificity for a specific method in a setting; longer lines imply greater imbalance and potentially greater bias in failure rate estimation. Background relatedness was set to 0.125.

Across the full range of simulations, classification was more accurate, as expected, when alleles were more likely to be detected and when transmission was lower, due to lower COI (Fig. 3). However, we found that Aster had consistently higher overall and more balanced sensitivity and specificity than either match-counting algorithm. Aster performance improved with larger, more informative panels, with 6 or more loci yielding excellent accuracy in moderate transmission and 12 or more loci in most high transmission settings. Even in simulations where recurrences were the most challenging to classify, such as high transmission settings with poor allele detection, the sensitivity and specificity of Aster remained high for the 18-locus panel and improved further with 48 loci (see Fig. 3). In contrast, while match-counting algorithms performed well in low transmission settings (with mostly monoclonal infections), the differences between sensitivity and specificity became pronounced at moderate and high transmission settings. This imbalance could lead to considerable overestimation or underestimation of overall failure rates, as explored below. Figure S1 provides similar comparisons but with varying background relatedness of infections and detection probability fixed at 0.9; as expected, higher relatedness led to decreased performance for match-counting algorithms. The performance of Aster was more robust, particularly when using larger panels.

In some cases, it may be more appropriate to include a distribution of background relatedness instead of a single value (e.g., when relatedness seems overdispersed or bimodal). As a real-world example of this type of distribution, we used the empirical distribution of pairwise relatedness obtained from D0 samples from a TES recently performed in Asayita, Ethiopia, using the MAD^4^HatTeR amplicon sequencing panel (24) (Fig. S2). We simulated pairs of infections with background relatedness drawn from this distribution and then classified them using Aster and match-counting algorithms; when a distribution was used as an input to Aster, the likelihood was calculated using numerical integration in equation 9. Aster provided accurate and balanced results, particularly when using more informative genotyping panels with larger numbers of diverse microhaplotype loci (Fig. S3). In contrast, match-counting algorithms gave poor results in moderate and high transmission settings for all genotyping panels.

### Study-level estimation accuracy

The results of a TES are interpreted at the study level, so we next simulated studies to evaluate the effect of classification accuracy on these results. First, we present the summary of the estimates obtained from studies simulated with different proportions of individuals with recrudescent and newly infecting strains. Each study had 100 individuals, a typical size for a TES. Proportions of individuals with new strains were set to 0.1, 0.3, and 0.5 for simulated settings of low, moderate, and high transmission intensity, respectively. For each combination of a genotyping panel, treatment failure rate, transmission intensity level, and probability of detection, 1,000 studies were simulated, and a failure rate was estimated as a proportion of individuals with recrudescences.

**Fig 4:**
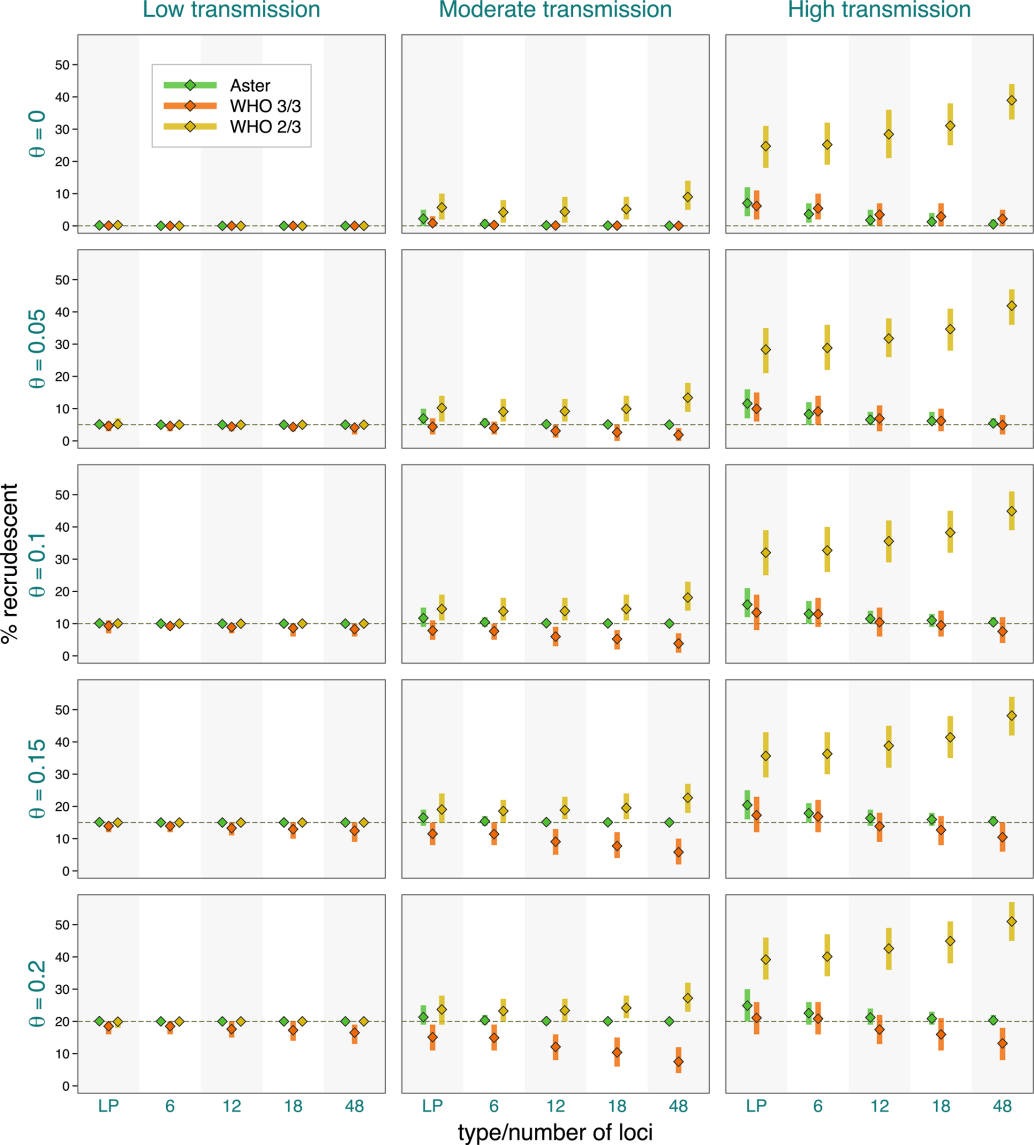
Study-level estimates across transmission intensity levels, genotyping panels, and true proportions (θ) of recrudescence events. The vertical bars represent a 95% range (0.025–0.975 quantiles) of the results, and the diamond symbols represent the mean. Detection probability was fixed at 0.9, and background relatedness was 0.

**Fig 5:**
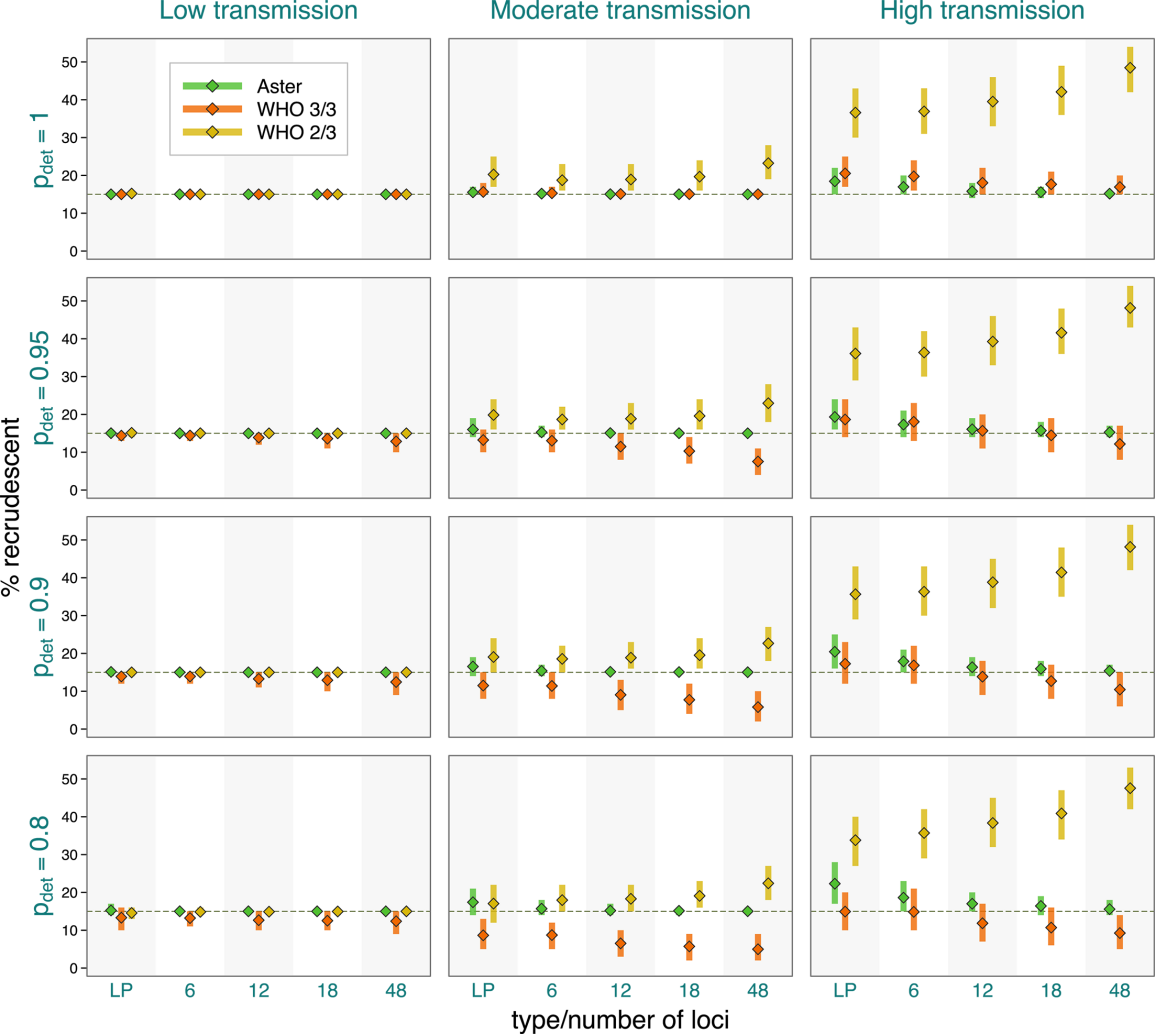
Study-level estimates across transmission intensity levels, genotyping panels, and detection probabilities ($p_{det}$). The vertical bars represent a 95% range (0.025–0.975 quantiles) of the results, and the diamond symbols represent the mean. Proportion of recrudescence events was fixed at 0.15, and background relatedness was 0.

As expected from classification results presented earlier, failure estimates based on Aster were the most accurate and were increasingly concentrated around the true proportion of recrudescence with greater numbers of loci (Fig. 4). For example, in the most challenging high transmission setting, when the true failure rate was 0.15, Aster produced results between 0.14 and 0.18 95% of the time with an 18-locus panel. In contrast, match-counting algorithm estimates were more biased, with the extent and direction of bias depending on transmission setting, true failure rate, genotyping panel, and algorithm. In general, higher transmission resulted in greater estimate variability, more bias, and greater differences between the estimates obtained with the two algorithms; notably, more informative panels led to greater bias. The 2/3 algorithm tended to overestimate failure, with dramatic overestimation in high transmission simulations, e.g., producing estimates between 0.28 and 0.41 95% of the time for a true failure rate of 0.05 when using 18 loci. The 3/3 algorithm underestimated the failure in moderate transmission, with more biased results for higher failure rates, e.g., with 95% of the estimates between 0.06 and 0.15 for a true rate of 0.2 with an 18-locus panel; the estimates were expectedly higher for high transmission, where the bias went in either direction. To evaluate the effect of imperfect allele detection on study results, we next fixed the proportion of recrudescences at 15% and considered a range of detection probabilities (Fig. 5). Expectedly, all the methods had excellent performance when allele detection was perfect in low transmission settings. In more challenging settings, Aster results remained close to the true values (particularly for larger microhaplotype panels) for all levels of missingness, while match-counting estimates shifted downward with lower detection probabilities as more true allele matches were missed.

While for panels with more than three loci, 2/3 and 3/3 algorithms are interpreted as thresholds for a proportion of the loci with allele matches (41), we also explored other thresholds within this range (Fig. S4). With results from these intermediate thresholds falling between those from the 2/3 and 3/3 algorithms, they were accurate for some combinations of settings but not for others. To more fully represent potential transmission settings, we separated COI distribution from the proportion of individuals with newly infecting strains (Fig. S5). Three diagonal panels in Fig. S5 correspond to low, moderate, and high transmission settings in Fig. 4 when θ=0.15.

**Fig 6:**
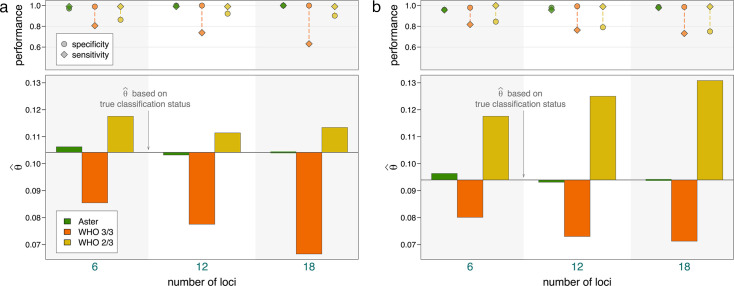
Therapeutic failure rate estimates ($\hat{\theta }$) with PK/PD model simulations using an amplicon sequencing panel with 6, 12, or 18 loci. For each figure, the bars in the lower panel represent deviation from the estimate based on true classification status; the upper panel displays performance measures. (**a**) Simulated data set from moderate transmission: 1,000 individuals with a mean COI of 3.5 for D0, incidence of 6 infections/year, and an average of 2.5 coinfecting strains per new infection. (**b**) Simulated data set from moderate transmission: 1,000 individuals with a mean COI of 5 for D0, incidence of 12 infections/year, and an average of 4 coinfecting strains per new infection.

To better approximate the underlying biological and ascertainment processes of a TES, we used a previously developed PK/PD model to simulate the dynamics of parasite clones present in D0 infections, new infections acquired during the 28-day follow-up period, and the effect of antimalarial treatment with artemether-lumefantrine (Fig. S6). For each simulated infection where recurrence would be detected, genotyping data were generated with detection based on relative and absolute parasite density (Fig. S7). We then applied genotyping classification algorithms to these data and calculated failure rates using the Kaplan-Meier survival estimator. The results are presented for two simulations with moderate and high transmission settings, respectively, each with 1,000 individuals. Comparison results were qualitatively similar to the simple simulations presented above where estimates were accurate for Aster but biased for both match-counting algorithms (Fig. 6). In both simulations, the 3/3 method consistently underestimated, while the 2/3 method consistently overestimated failure rates. Underestimation with the 3/3 method was more pronounced in the moderate transmission setting (producing an estimate of <0.07 using 18 loci when the true failure rate was >0.10), and overestimation with the 2/3 method was more pronounced in the high transmission setting (producing an estimate of >0.13 using 18 loci when the true failure rate was <0.10). This trend is consistent with previous results and is a likely consequence of more alleles matching by chance in the high transmission setting due to higher COI. Also consistent with previous simulation results, Aster outperforms match-counting algorithms in individual recurrence classifications (reflected in the top panels of Fig. 6).

### Input estimation, parameter misspecification, and assumption violations

**Fig 7:**
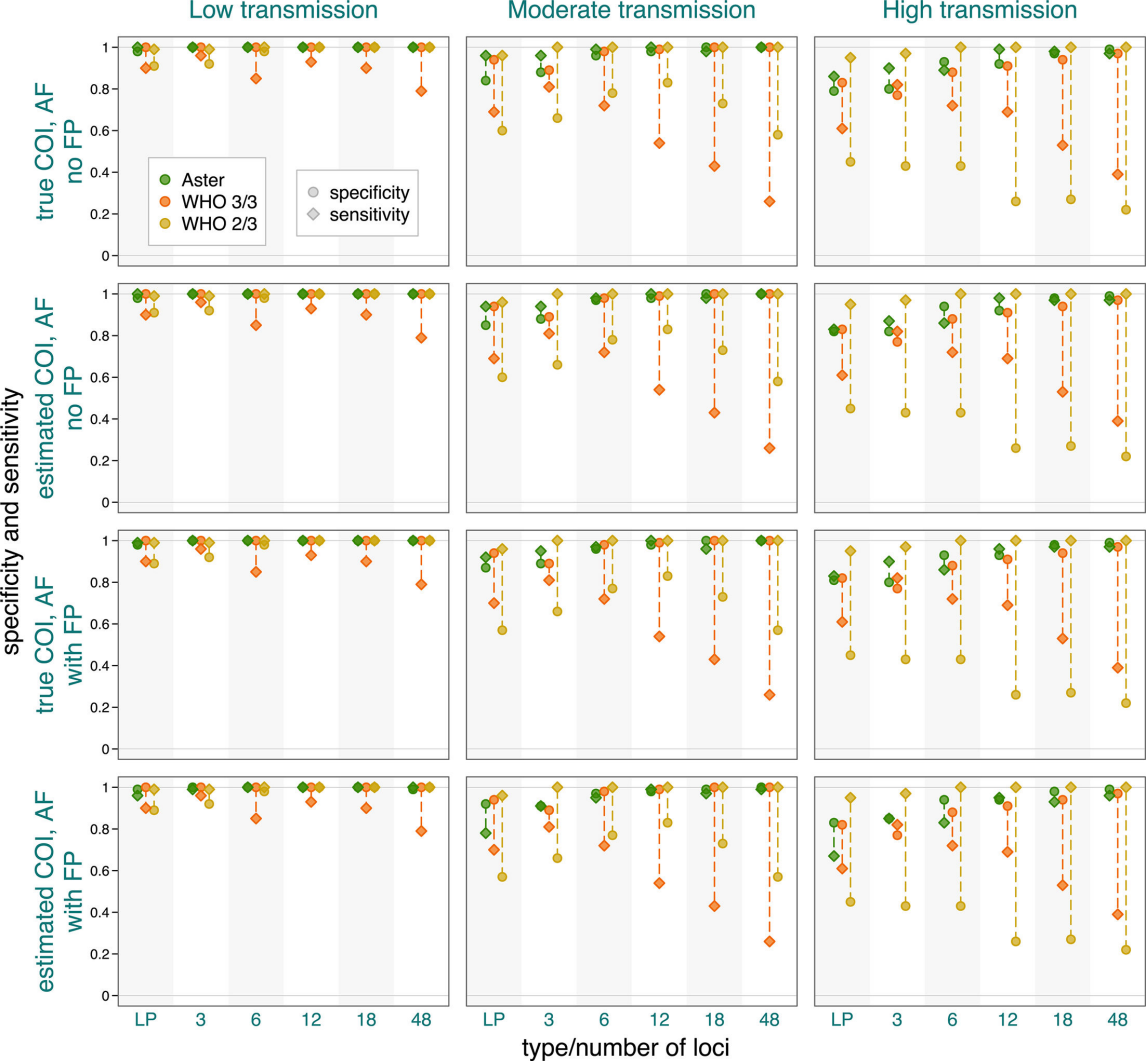
Sensitivity and specificity of Aster and match-counting algorithms exploring the effect of using estimated vs true COIs and population allele frequencies (AFs) and adding simulated false positive alleles (FPs). Allele frequencies were estimated from 100 D0 samples. False positive rates were 0.1 for the length polymorphism panel and 0.02 for the amplicon sequencing panels. Detection probability was fixed at 0.9, and background relatedness was 0.125.

Since COI, population allele frequencies, detection probabilities, and other Aster model parameters are not usually known in practice and are estimated, inaccuracies of such estimates might affect Aster results. Therefore, we explored Aster’s sensitivity to misspecifications by testing the method with different scenarios. Estimating COI and allele frequencies from simulated data had a minimal effect on Aster results (Fig. 7, rows 2 and 4). Simulation of false positive alleles, though not formally included in the estimation process, had a minimal effect on amplicon sequencing results but did decrease accuracy for length polymorphisms, which were simulated with a higher false positive rate (Fig. 7, rows 3 and 4). Additional simulations indicated that false positive alleles appearing at rates consistent with modern amplicon sequencing do not meaningfully affect Aster’s performance (Fig. S8). COI misspecification can have a moderate effect on the results, but this effect decreases for more informative panels (Fig. S9). A tuning parameter regulating potential false positive alleles was set dynamically to minimize the effect of COI misspecifications and address a large number of alleles assumed to be false positives when input COI is “underestimated.”

Sensitivity to misspecification of allele detection probability is of particular importance, since perfect detection is unlikely and missingness is generally difficult to estimate in practice. To assess the effect of misspecification, we simulated studies of 100 individuals and looked at the distribution of failure rate estimates obtained with different detection probabilities in a recrudescent strain in a D0 sample (Fig. S10). Assuming perfect detection resulted in considerable underestimation when true detection probability was less than 1, but the effect of other misspecified values was much smaller. For example, at a true value of 0.7 allele detection (fairly poor sensitivity), assumed values between 0.8 and 0.9 still resulted in estimates of treatment failure within 0.012 and 0.018 of the true value on average; assuming perfect detection shifted the values by nearly 0.15. Thus, in all the cases when true detection was not perfect, specifying a reasonable estimate of detection probability resulted in more accurate classification than assuming no missingness at all.

## DISCUSSION

Results from TES inform national and regional antimalarial drug policies. For these results to be reliable, genotype-corrected outcomes should be consistently accurate across transmission intensities and choice of genotyping assay. Our results indicated that match-counting algorithms provide accurate failure rate estimates only if used with a threshold appropriate for a specific combination of transmission settings, genotyping panel, and other factors. Identifying such a threshold would require *a priori* knowledge that might be challenging to obtain, require extensive efforts, and may still be prone to biased results. In contrast, as a principled statistical framework, Aster delivers accurate results in any setting without prior knowledge or arbitrary decision making. By explicitly accounting for population allele frequencies, COI, background relatedness, and allele detection probability, Aster identified recrudescence events with high sensitivity and specificity, reliably estimating therapeutic failure rates across a range of settings. Furthermore, as data from larger, more informative genotyping panels were included, Aster estimates became more accurate—a feature that will become increasingly useful as genotyping methods continue to evolve (24, 41–44).

Comparison between Aster and match-counting algorithms across different settings provided insight into where WHO-recommended match-counting algorithms would be likely to fail and where using Aster would be most advantageous. When both failure rate and transmission intensity are low, and there are few recurrent infections to classify, the choice of method does not make much of a difference. Similarly, when COI is low and loci are highly diverse, classification is accurate with either approach. The difference between methods increases for lower diversity panels, where the probability of alleles matching by chance is higher. As COI got higher, the difference between Aster and match-counting algorithms became greater and was particularly salient in data simulated from high transmission areas—such as those where partial artemisinin resistance is now spreading and where outcome classification is more important and more challenging. Probability of allele detection affected match-counting algorithms, and higher background relatedness decreased their performance as well.

Since Aster incorporates important factors that meaningfully impact estimates of treatment failure, we consider the practical implications of connecting upstream data and external information to Aster methodology and using them as Aster inputs. The COI of infections varies with malaria transmission intensity, affecting the number of alleles matching by chance and consequently the results of traditional match-counting algorithms (9, 10, 12). While the true COIs of samples are unknown, they can be readily estimated from empirical data and incorporated into Aster to minimize bias in outcome classification. Similarly, allele frequencies will vary based on the loci genotyped and the local parasite population, affecting the probability of alleles matching. These too can be readily estimated from empirical data, for example, using all or a random subset of D0 samples. Information on background relatedness between infections is explicitly included in Aster since underestimating the relatedness in a local population can lead to misclassifying a new infection as a recrudescence. As with allele frequencies, an estimate of background relatedness can be obtained from D0 samples, for example, by estimating their pairwise IBD proportion, and supplied as an input to Aster. When the number of loci in a panel is relatively small, individual pairwise relatedness estimates may have high uncertainty (31), but with a sufficient number of D0 samples, relatedness summary estimates (e.g., mean) should still be reasonably accurate. Therefore, special consideration should be given to the number of genotyped D0 samples used to estimate population allele frequencies and background relatedness (45). Imperfect detection of alleles may also affect results but is more difficult to estimate, potentially varying with genotyping method, COI, and parasite density. Fortunately, we show that Aster is robust to misspecification of detection probability as long as detection is not assumed to be perfect. Thus, including imperfect detection in the framework calibrates missingness in a principled way, allowing Aster to correctly classify recurrence as a recrudescence when recrudescent strains are not fully detected and would likely be missed by the 3/3 algorithm. Another type of genotyping error, false positive alleles, is not formally included in the current framework. However, while false positives can have an effect on the accuracy of match-counting algorithms, Aster is based on an IBD approach, which we demonstrate minimizes the effect of false positives on classification accuracy.

One of the advantages of the Aster framework is its modular design with explicit assumptions, where individual components can be changed to reflect additional knowledge and updated assumptions or adapted to developing technology. New components that model additional biological and genotyping processes can be added to the framework, allowing it to improve accuracy by utilizing external information. For example, components that deal with completely undetectable strains or persistence of gametocytes could be included, potentially correcting the estimates and especially the uncertainty, which would reflect the amount of unknowable information more realistically. The framework design also affords greater flexibility in terms of inputs (such as strain- and locus-specific probability of detection, using input distribution instead of a single value, and encoding prior information) and inferential approaches accommodating the knowledge that might become available. A formal framework allows Aster to approach the issue of missing data (no detected alleles at a locus, missing samples, or failed genotyping) in a systematic way. In turn, this can inform a principled treatment of right censoring and missingness in a failure rate estimation, regardless of the estimator used. For example, a recurrence with no genotyping data needs to be treated differently than a voluntary withdrawal from the study at the same time point: an individual with a recurrent infection would be more likely to have experienced a recrudescence by the end of the follow-up had they completed the study than an individual with loss of follow-up without a recurrence since a conditional probability of recrudescence given recurrence is greater than a marginal probability of recrudescence.

In our simulations, we explore Aster results in a variety of settings that include COI, detection probability, and background relatedness. For genotyping panels, we vary the type of panel and number of loci, but all synthetic amplicon sequencing panels are based on a panel with population allele frequencies obtained from sub-Saharan Africa (within which the currently used locus diversity is similar) (24). In practice, locus diversity may vary depending on the population and choice of genotyping panel. While we did not vary locus diversity for simulations in this paper, we provide guidance on the number and diversity of loci needed to achieve desired classification accuracy in relation to COI elsewhere (Table 4 in reference 45).

Aster will need to be evaluated in TES conducted in various geographic settings and with various genotyping methods to confirm its utility in real-world settings. While designed for TES, Aster can be applied to relevant studies such as regulatory trials of new antimalarial drugs or longitudinal studies where determining the presence or absence of persistent clones would be of interest. It can also accommodate TES with different designs and additional genotyped samples from intermediate time points.

Reliable assessment of antimalarial efficacy is more pressing than ever, with resistance spreading in high transmission areas where accurate genotyping correction is the most challenging. Our results suggest that accuracy can be greatly improved through the application of genotyping tools that provide rich genetic data, such as sensitive, diverse amplicon sequencing panels (24, 46), combined with analytical methods that take full advantage of these data and deliver consistent results across transmission levels. Fortunately, access to next-generation sequencing has dramatically improved; over 80% of national public health labs in sub-Saharan Africa now have technology in place to generate high-quality genotyping data (Africa CDC, personal communication, and [47]). Taking advantage of these data requires principled statistical approaches in place of simple matching-counting algorithms that can provide inconsistent results, which vary by genotyping panel and epidemiological setting. Aster provides a structured statistical framework that is able to accommodate various types of information and can be amended with additional features in concordance with evolving laboratory methods and bioinformatic pipelines. The *asterTES* package, which currently provides recurrence classification with corresponding posterior probabilities and a failure rate estimate using the Kaplan-Meier survival estimator, features a high level of flexibility for the user and can be extended along with the framework. With its fast and user-friendly implementation, Aster can contribute to accurate assessment of antimalarial TES, aiding stakeholders in timely determination of effective management of malaria.

## SUPPLEMENTAL MATERIAL

Supplemental material aac.01411-25-s0001.pdf Fig. S1 to S10.

ASM does not own the copyrights to Supplemental Material that may be linked to, or accessed through, an article. The authors have granted ASM a non-exclusive, world-wide license to publish the Supplemental Material files. Please contact the corresponding author directly for reuse.
